# The alkalophilic fungus *Sodiomyces alkalinus* hosts beta- and gammapartitiviruses together with a new fusarivirus

**DOI:** 10.1371/journal.pone.0187799

**Published:** 2017-11-29

**Authors:** Lenka Hrabáková, Alexey A. Grum-Grzhimaylo, Igor Koloniuk, Alfons J. M. Debets, Tatiana Sarkisova, Karel Petrzik

**Affiliations:** 1 Department of Plant Virology, Institute of Plant Molecular Biology, Biology Centre of the Czech Academy of Sciences, České Budějovice, Czech Republic; 2 Faculty of Science, University of South Bohemia in České Budějovice, České Budějovice, Czech Republic; 3 Laboratory of Genetics, Wageningen University, Wageningen, The Netherlands; University of Texas Medical Branch at Galveston, UNITED STATES

## Abstract

Mixed infection by three dsRNA viruses, a novel betapartitivirus, a gammapartitivirus, and a novel fusarivirus, has been identified in four isolates of the obligate alkalophilic fungus *Sodiomyces alkalinus*. The first, Sodiomyces alkalinus partitivirus 1 (SaPV1), is placed within the genus Betapartitivirus and is related to Ustilaginoidea virens partitivirus 2. The taxonomic position of the second virus is less clear as it shares high (85%) amino acid sequence identity but significantly low (77%) nucleotide sequence identity of the capsid protein with Colletotrichum truncatum partitivirus 1. The third, the novel Sodiomyces alkalinus fusarivirus 1 (SaFV1), is related to Fusarium poae fusarivirus 1. All the viruses show efficient vertical transmission through asexual and sexual spores. These novel coexisting viruses do not evoke apparent phenotypic alteration to their fungal host. This is the first description of a viral infection in an alkalophilic fungus.

## Introduction

Mycoviruses are widespread in fungi, and can carry various types of nucleic acids that constitute their genomes. Most of the described mycoviruses possess dsRNA and positive-sense ssRNA genomes, however some ssDNA-, dsDNA-, and negative-sense ssRNA-viruses have also been reported [1–3]. The ssRNA mycoviruses are assigned to at least 12 families, while the dsRNA mycoviruses are currently ascribed to seven families (*Partitiviridae*, *Reoviridae*, *Megabirnaviridae*, *Totiviridae*, *Chrysoviridae*, *Endornaviridae*, *Quadriviridae*). Furthermore, a large number of ssRNA and dsRNA viruses have been described, but remain unclassified. It is believed that mycoviruses lack an extracellular phase in their lifecycles, and are not infectious as free particles. However, in case of Sclerotinia sclerotiorum hypovirulence-associated DNA virus 1 (SsHADV1), the purified particles were infectious and the virus could be transmitted extracellularly [4]. In addition, mycoviruses were detected in two soil-inhabiting fungivorous oribatid mites (Acari: Oribatida) and mites (Acari: Acaridae) which could potentially serve as vectors [5–6]. Recently, the mycophagous insect *Lycoriella ingenua* (Diptera: Sciaridae) has been found to be infected with the mycovirus SsHADV1. Viruliferous adults of this insect could transmit the virus transovarially [7]. Mycoviruses can be transmitted horizontally via hyphal anastomosis between genetically compatible fungal strains and vertically through spores. Vertical transmission of mycoviruses in *Ascomycetes* occurs by asexual spores and, more rarely, by sexual spores, while in *Basidiomycetes* mycoviruses are usually passed through sexual spores [8, 9]. A virus is effectively immortal within its host, as there is no known way for the fungus to eliminate the virus, since intracellular incompatibility between different viruses and their respective hosts has not been described. Mycoviruses often display complex spectra of dsRNA in the nucleic acid samples viewed on the gels, which is suggestive of multiplex viral infections [10–11]. Most mycoviruses described to date appear to have no effect on their hosts. However, mycovirus infection in natural isolates of *Aspergillus niger* resulted in reduced competitive fitness, and, in some cases, phenotypic alterations [12]. In some plant-pathogenic fungi hypovirulence is caused by ssRNA hypoviruses, mitoviruses, dsRNA reoviruses and totiviruses and ssDNA mycovirus [13–14]. Another well-known example is the killer phenotype of *Saccharomyces cerevisiae* yeast governed by the cytoplasmic dsRNA virus [15]. It has been argued that hypovirulence in plant pathogenic fungi is beneficial for the survival of both the virus and the fungal host, because hypovirulent fungal strains do not kill the plant hosts and can also modulate strains’ virulence [16]. Furthermore, there are indications that dsRNA of mycoviruses from families *Chrysoviridae*, *Endornaviridae*, *Partitiviridae* and *Totiviridae* that have co-evolved with their respective hosts for a long time, have been integrated into host genomes. Mycovirus-like sequences have been identified even in eukaryotic lineages, which were not expected to be hosts for these viruses, e.g. partitivirus-like sequences in monocots, flatworms, and sand fly; totivirus-like sequences in diatom, and animals; and chrysovirus-like sequences in plants [17–20]. Both mRNA transcripts of RNA-dependent RNA polymerase (RdRp) and capsid protein (CP) of integrated totivirus- and partitivirus-like genes appeared in the hosts, suggesting that the endogenous viral proteins have been co-opted for new cellular functions [19–21].

**Fig 1:**
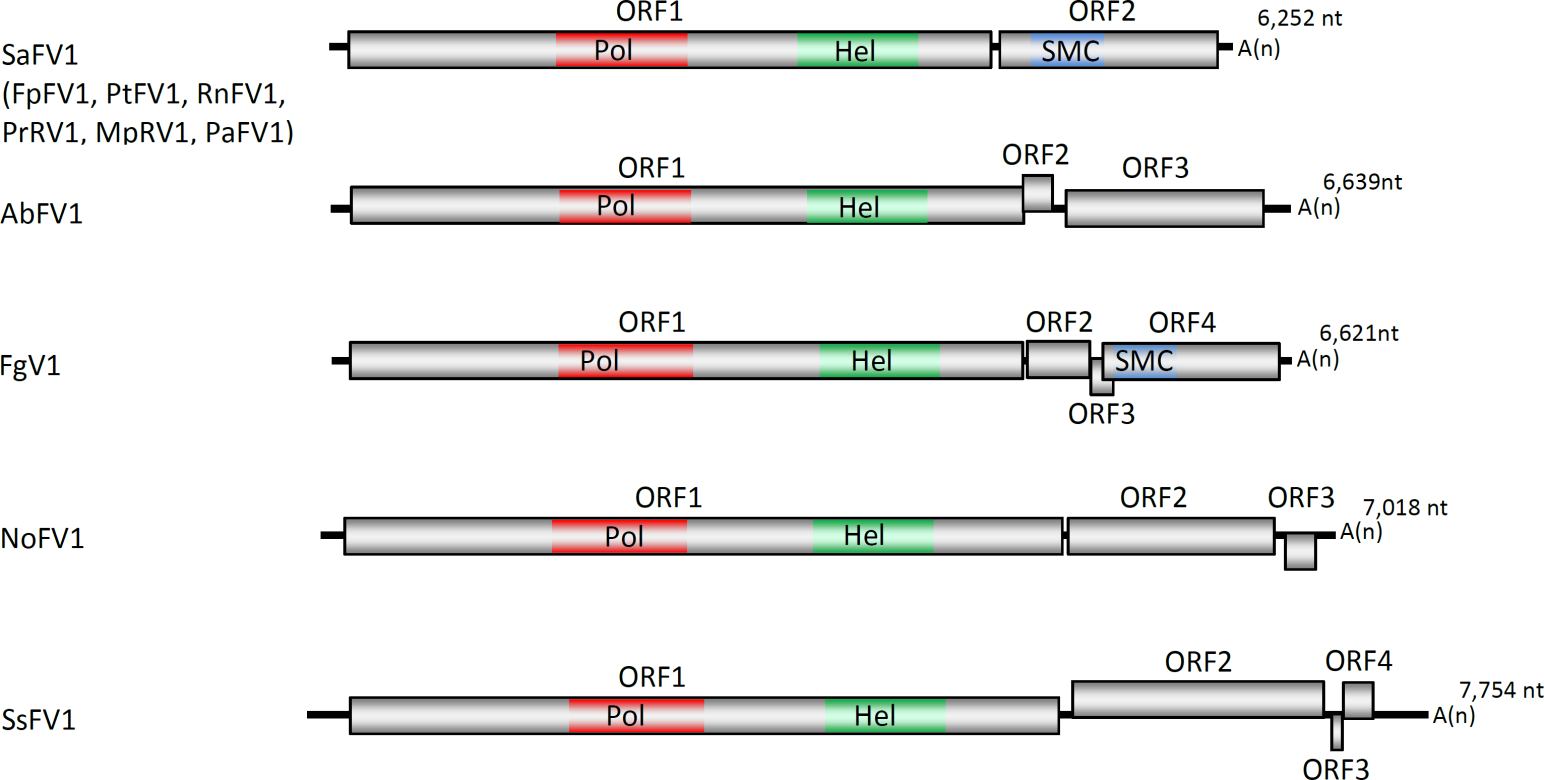
Schematic representation of the putative genomic organization of fusariviruses. The boxes and lines represent open reading frames (ORFs) and non-coding sequences, respectively. Positions of polymerase (Pol, red), helicase (Hel, green), and structural maintenance of chromosomes (SMC, blue) domains are indicated. Genomes are shown to scale. The A(n) represents poly (A) tail. Sodiomyces alkalinus fusarivirus 1 –SaFV1, Fusarium graminearum virus—FgV1, Sclerotinia sclerotiorum fusarivirus 1—SsFV1, Alternaria brassicicola fusarivirus 1- AbFV1,—Nigrospora oryzae fusarivirus 1—NoFV1. Fusarium poae fusarivirus 1 –FpFV1, Pleospora typhicola fusarivirus 1 –PtFV1, Rosellinia necatrix fusarivirus 1 –RnFV1, Penicillium roqueforti ssRNA mycovirus 1 –PrRV1, Macrophomina phaseolina ssRNA virus 1 –MpRV1, and Penicilium aurantiogriseum fusarivirus 1 –PaFV1, have similar genome organization as SaFV1.

Fusariviridae is a recently proposed family of mycoviruses with a positive polyadenylated ssRNA genome of 6–7.7 kb long and two large open reading frames (ORFs) (Fusarium poae fusarivirus 1 –FpFV1, Pleospora typhicola fusarivirus 1—PtFV1, Rosellinia necatrix fusarivirus 1 –RnFV1, Penicillium roqueforti ssRNA mycovirus 1 –PrRV1, Macrophomina phaseolina ssRNA virus 1 –MpRV1, and Penicilium aurantiogriseum fusarivirus 1 –PaFV1) and one (Alternaria brassicicola fusarivirus 1 –AbFV1, and Nigrospora oryzae fusarivirus 1 –NoFV1) or two (Sclerotinia sclerotiorum fusarivirus 1 –SsFV1, Fusarium graminearum dsRNA mycovirus 1 –FgV1) small ORFs on the coding strand [2] (Fig 1). RdRp and helicase (Hel) domains have been recognized on the longest ORF1. The product of the second largest ORF has no significant similarity with any known protein in GenBank: the most similar, sharing 27–30% identity, are the corresponding proteins of other fusariviruses. No conserved domains were detected in the motif library based on the small ORFs-encoded protein sequences. No CP or CP-like genes were found in the genome of fusariviruses, and no viral particles were seen in the infected hosts. About 10 more viruses have recently been grouped into this clade, and several partial fusari-like sequences have been detected by bioinformatics approaches in the transcriptome shotgun libraries of public databases [22]. All known fusariviruses are from the ascomycetous fungi of classes *Dothideomycetes*, *Leotiomycetes*, and *Sordariomycetes*. Virulence varies considerably among fusariviruses. For instance, FgV1 is responsible for reducing mycotoxin production and virulence of *F*. *graminearum* strain DK21 [23], while RnFV1, SsFV1, and AbFV1 infect natural host asymptomatically [2, 22–24].

Partitiviruses (family *Partitiviridae*), contrary to fusariviruses, are bi-segmented dsRNA viruses that infect plants, fungi, or protozoa [25]. They possess two essential dsRNA genome segments that are encapsidated separately and contain one ORF each. Partitivirus particles are isometric, measuring 25–40 nm in diameter and consist of 120 CP molecules, one RdRp molecule, and one dsRNA segment. These viruses pack both ssRNA replication intermediate and genomic dsRNA into virions but only particles containing dsRNA are transcriptionally active [26–27]. The RNA 1 segment encodes the RdRp protein, and the RNA2 segment encodes the CP. Partitiviruses are transmitted vertically during cell division or horizontally by anastomoses between fungal hyphae. They mediate persistent infections of their hosts and have a few, if any, deleterious effects on the host [25]. One exception is Rosellinia necatrix partitivirus 6 which induced profound phenotypic alterations in *Cryphonectria parasitica* characterized by reduced growth rate and enhanced pigmentation [28]. Even though hypovirulence is not a common phenomenon in partitiviruses, Sclerotinia sclerotiorum virus 1 does cause hypovirulence in *Sclerotinia* spp. and *Botrytis cinerea* [29]. Also, Rhizoctonia solani partitivirus 2 reduces mycelial growth and confers hypovirulence in the basidiomycetous fungus [30]. Some Heterobasidion partitiviruses diminish growth rates of both natural and acquired hosts at lower temperatures but not at elevated temperatures [31]. Partitiviruses constitute the following genera: *Alphapartitivirus* from fungi and plants (14 species), *Betapartitivirus* from fungi and plants (17 species), *Gammapartitivirus* from filamentous fungi (8 species), *Deltapartitivirus* from plants (5 species), and *Cryspovirus* (1 species from protist in a recent taxonomy) [32]. Furthermore, recent metagenomic analyses of mammalian, raw sewage, and freshwater lake samples have identified additional sequences attributable to novel partitiviridae members [25]. The key criteria for partitivirus classification are host range, size difference of the genome segments and/or encoded proteins lengths, and protein sequence similarity.

In this study, we describe dsRNA viruses nested in *Sodiomyces alkalinus*, a soda-lake fungus with an unusual ability to grow optimally at very high ambient pH [33]. We show that geographically distant isolates contain a unique set of two partitiviruses and one fusarivirus. We discuss the evolutionary forces driving the virus variability and stability in the host.

## Material and methods

### Fungal strains

**Fig 2:**
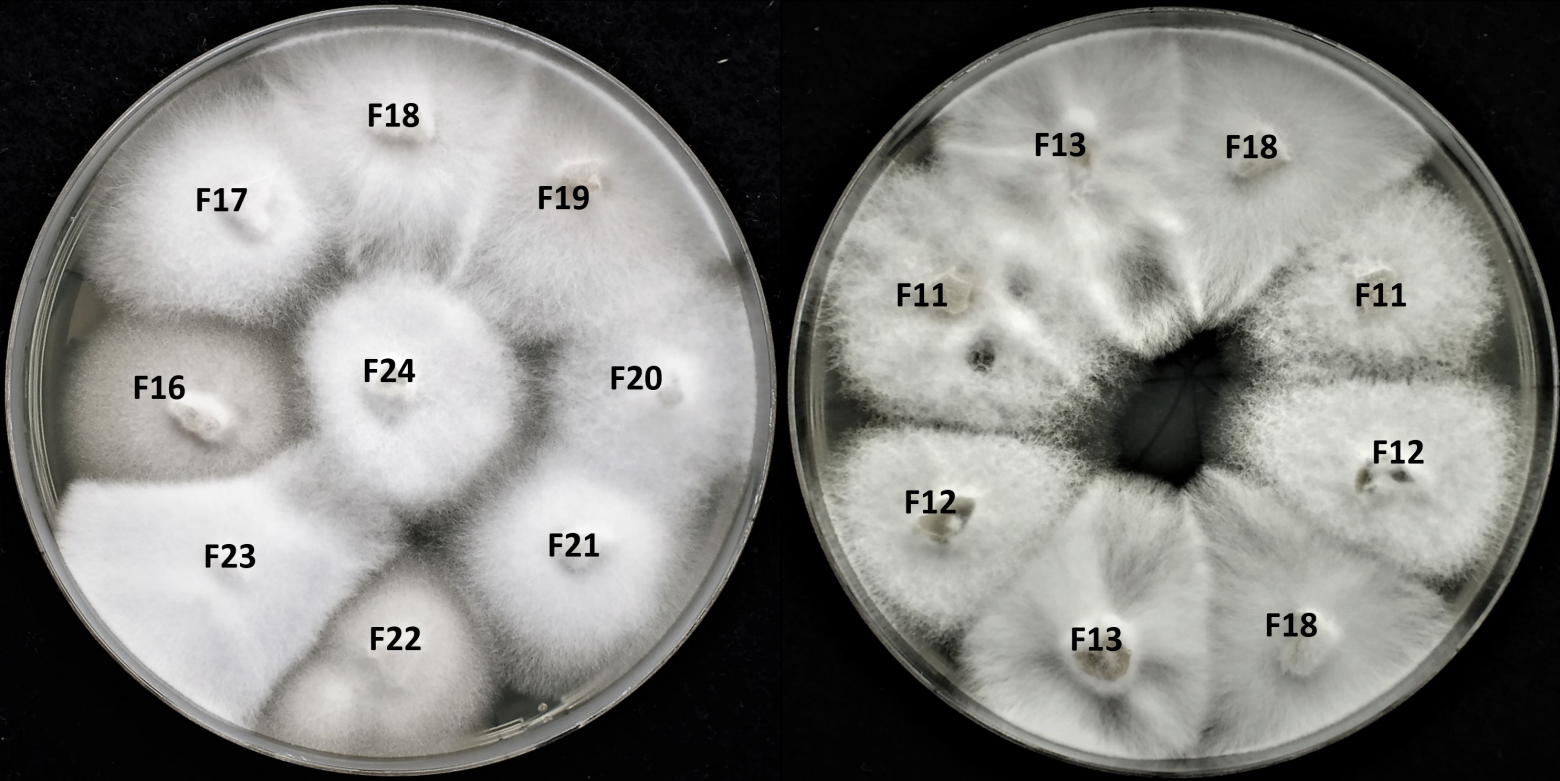
*S*. *alkalinus* isolates growing on alkaline agar plate. Five days old isolates of *S*. *alkalinus* did not form inhibition zones or barrages between the strains although the mycelia did not fuse with each other.

All 18 known strains of *S*. *alkalinus* isolated previously [26] were used in the current study. Routine sub-culturing was performed using the alkaline agar (AA, pH 10) medium containing per liter: Na_2_CO_3_−24 g, NaHCO_3_−6 g, NaCl– 5 g, KNO_3_−1 g, K_2_HPO_4_−1 g, malt extract– 17 g, yeast extract– 1 g, and agar– 20 g. The cultivation was at 27°C in the dark. To obtain colonies derived from single ascospores, cleistothecia were picked manually, rolled on the surface of water agar (2%) medium to remove residual mycelium, and then placed in 0.09% saline/0.005% Tween-80 solution and gently crushed. The released ascospores were plated onto the AA medium and young colonies were transferred to fresh AA plates. No inhibition or barrages were observed in the interaction zone between the strains grown next to each other although the mycelia did not fuse with each other (Fig 2). Hyphal tips cultivation on AA plates with 355 μM of cycloheximide was used to cure the isolate F13 of viruses.

### Isolation of nucleic acids and viral particles

Double-stranded RNA was isolated from about 0.1 g of wet mycelium after 10 days of cultivation on AA medium by the CF-11 cellulose chromatography method, as described by Morris and Dodds [34]. The isolated nucleic acids were S1 nuclease and DNase I treated, and separated on agarose gel.

The viral particles were isolated from the mycelium crushed with a pestle in a mortar, resuspended in 0.1 M phosphate buffer and clarified by centrifugation at 10 000 rpm. Then pelleted from the supernatant by ultracentrifugation at 30 000 rpm for 3 h. The resuspended pellet was negatively stained with phosphotungstic acid and observed with a JEM1011 transmission electron microscope.

For relative quantification of viruses, total RNA was isolated using a NucleoSpin RNA Plant kit (Macherey Nagel, Düren, Germany) from *S*. *alkalinus* grown on AA medium. Complementary DNA (cDNA) was prepared with Superscript IV (Invitrogen) following manufacturers’ instruction, and random hexamer primer. Relative concentrations of viruses were estimated by real-time reverse-transcription polymerase chain reaction analysis (RT-PCR). For SaFV1, SaPV1, and CtParV1-SAL, specific primers (5´-3´) SaFV1-1902f: GACATTTACCGAAAAGGAACT, SaFV1-2333r: CATAGACTTCACCTTGCTCA, SaPV1-405f: GCTCAACACATCATGAACAA, SaPV1-804r: CATCTATGCGTTCGTTAAGC, CtParV1-SAL-810f: CTTCTTGGTGTGGAGATCAA, and CtParV1-SAL-1181r: AGCAGCATTTCTATTGACCA were used.

### Northern blot

Northern blot analysis was conducted as described in [35]. Total RNA obtained from the isolates F7 and F13 were resolved on an agarose gel in TAE buffer, denatured by soaking the gel in 50 mM NaOH for 30 min and neutralized with Tris-HCl (pH 7.5). The RNAs were transferred onto a nylon membrane by capillary blotting with 2xSSC and crosslinked to the membrane by UV irradiation. DIG-labelled probe specific for SaFV1 nucleotide sequence spanned map positions 5026–5412 was amplified by primers 5´-TGGACCTTAAAAATGCTTGG-3´ and 5´-GTGCCTTCAGTACATTAGCA-3´ and prepared with the PCR DIG labelling kit (Roche Diagnostics, Mannheim, Germany). Detection was performed using alkaline phosphatase-conjugated, anti-DIG antibody (Roche) and DuoLuX chemiluminescent/fluorescent substrate (Vector Laboratories, Burlingame, CA, USA). Viral bands were detected by fluorescence.

### Next-generation sequencing and data analysis

Sequencing libraries were prepared from the dsRNA template using TruSeq RNA Library Preparation kit (for the isolates F11 and F12) or MuSeek library preparation kit for Illumina (Thermo Scientific) (for the isolates F13 and F18) and then sequenced with a HiSeq 2500 ultra-high-throughput sequencing system in SE 100bp mode (GATC Biotech, Germany and SEQme, s.r.o., Czech Republic). The reads were trimmed and assembled in CLC Genomics Workbench 8.5.1 (Qiagen, Denmark). Contigs were cured, gaps between them were filled by amplification with specific primers, and Sanger sequenced. Terminal sequences were obtained using the 5´ and 3´ RACE System (Invitrogen) and virus specific primers (S1 and S2 Tables).

Sequence similarity searches were conducted using BLAST programs in the NCBI database. Multiple sequence alignments were carried out using the CLUSTALx [36] and MEGA v.7 programs [37]. Maximum likelihood analysis was used to infer virus phylogeny with a 1000 bootstrap replicates. Sequence polymorphisms were analysed using the DnaSP v5 program [38]. Sequence secondary structures were calculated using the RNAfold program on RNAfold WebServer on the web page http://rna.tbi.univie.ac.at.

Quantities of dsRNA amount were compared as following: dsRNA was separated on an agarose gel and stained with SYBR Green. The stained RNA was observed using Kodak EDAS 290 system and relative intensity of dsRNA bands, expressed as integrated area of the peak, was compared. Results were averaged from the three independent dsRNA isolations.

## Results

### Strains and virus presence

**Fig 3:**
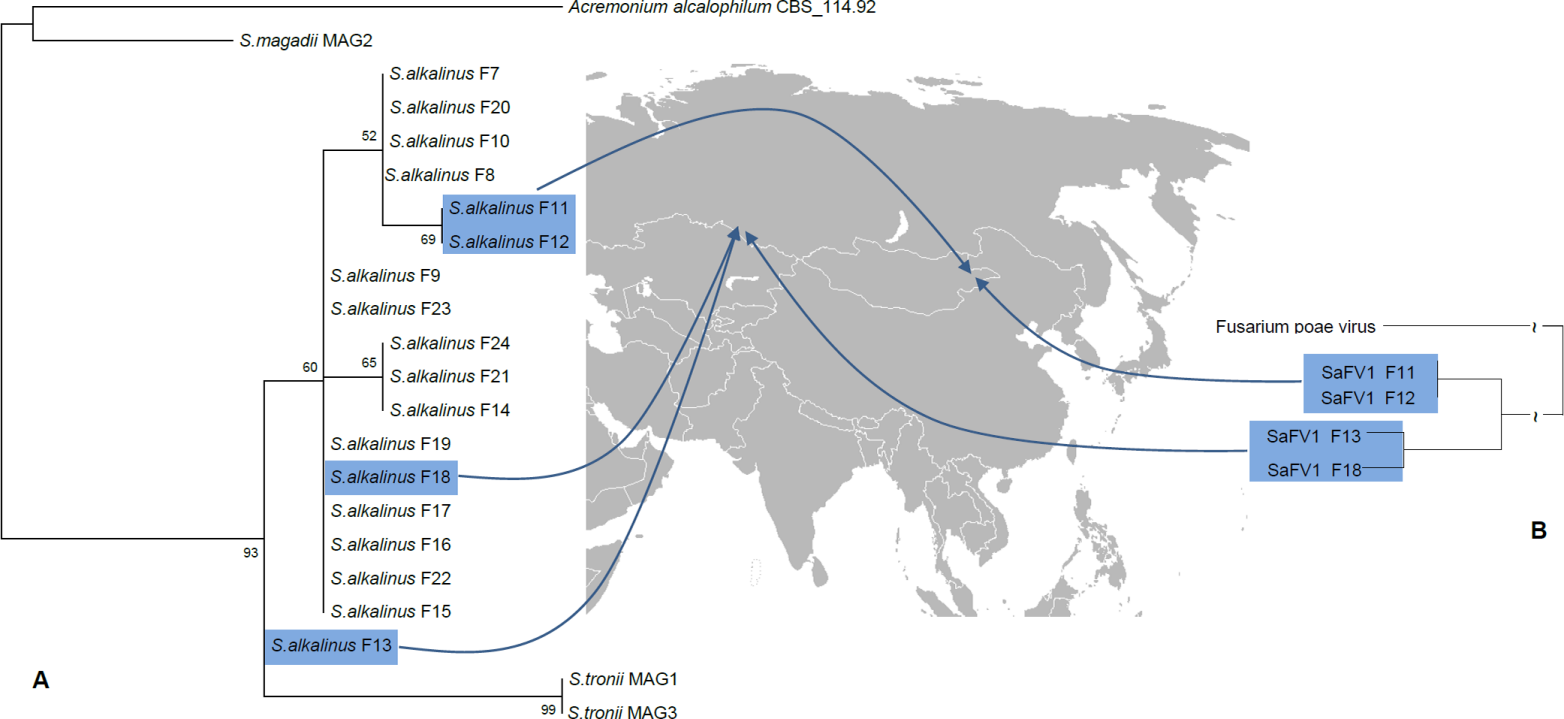
Phylogeny of the *Sodiomyces* sp. based on the ITS rDNA sequences (A), with the origin location of dsRNA-containing strains and phylogeny of the SaFV1 isolates based on the whole genome sequence (B).

**Fig 4:**
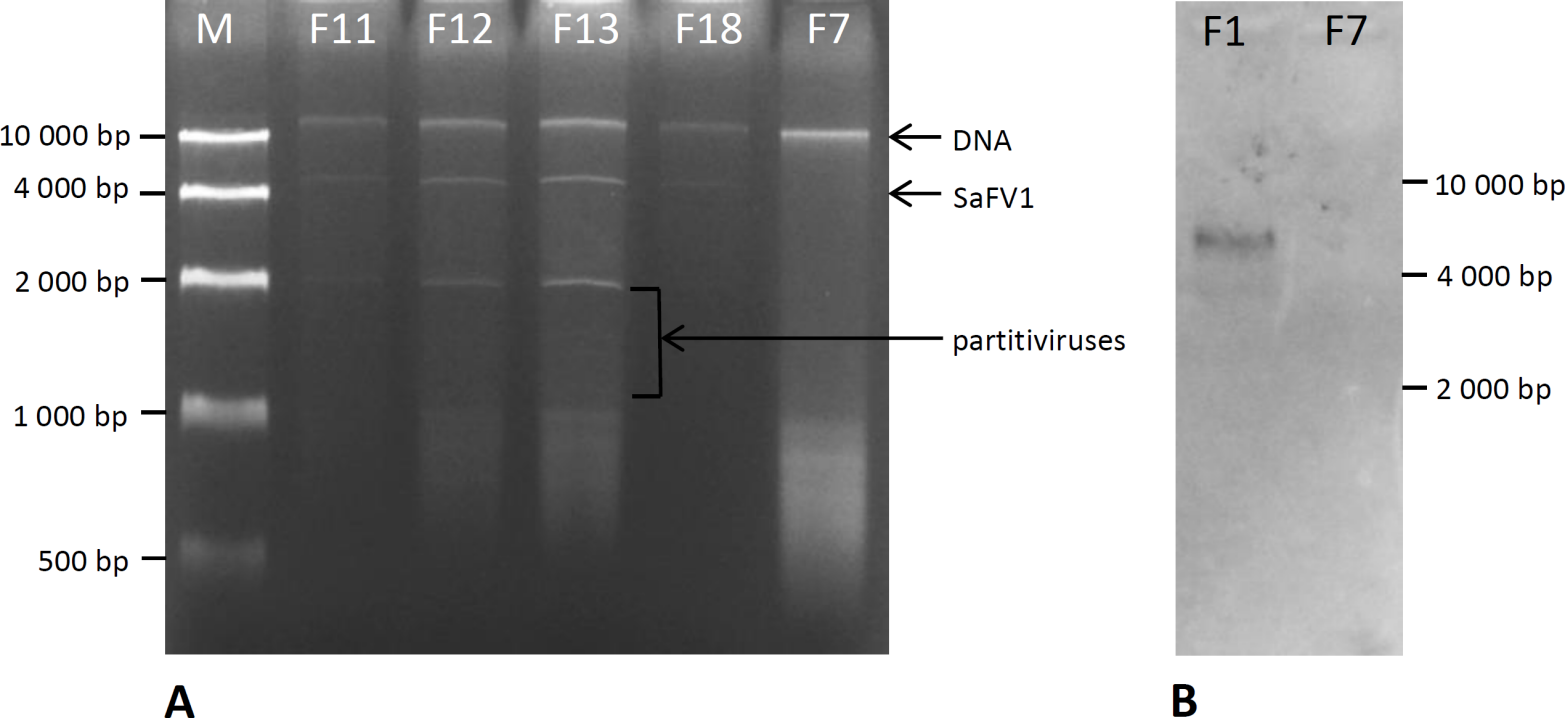
**A**) Double-stranded RNA profile of the virus-containing isolates F11, F12, F13, F18, and a virus-free isolate F7 of *S*. *alkalinus*. Viral dsRNAs are marked with arrows. M–DNA size standard. **B**) Detection of SaFV1 in isolates F13 and F7 by total RNA extraction and Northern blot analysis. Total RNA from virus-containing and virus-free isolates was extracted and resolved by electrophoresis in TAE gel, transferred to a nylon membrane, and probed with specific DIG-labelled probe corresponding nt positions 5026–5412 for SaFV1. The band of about 6.2 kb in size that match the SaFV1 genome size and faint band <4kb indicating the presence of subgenomic RNAs were detected (highlighted by arrows).

**Fig 5:**
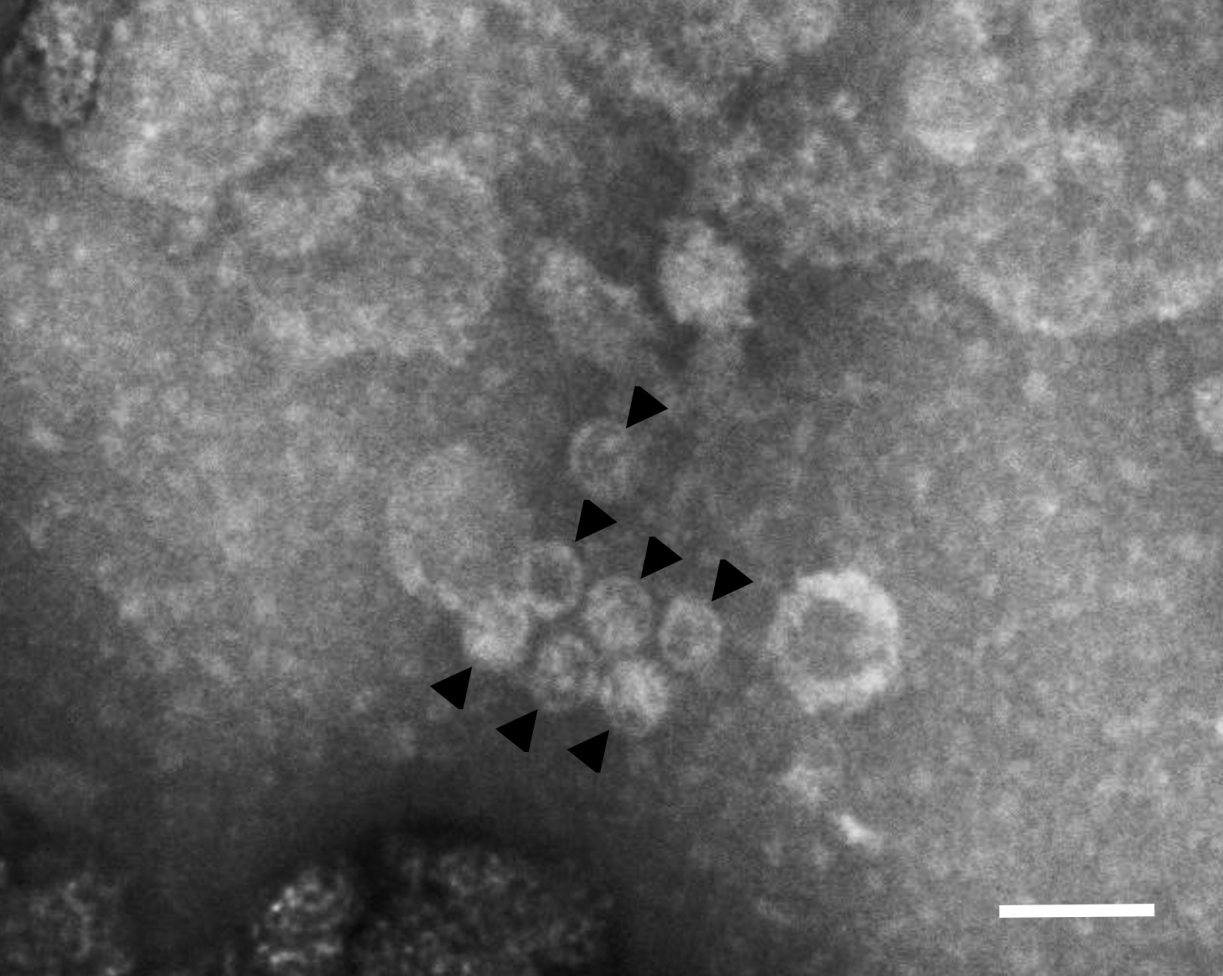
Negative stained virus-like particles (highlighted by arrows) in the cellular extract of the *S*. *alkalinus* strain F13. Scale bar = 100 nm.

Four strains of *S*. *alkalinus* out of the known 18 contained detectable amounts of high-molecular weight dsRNA. These strains were collected in northeast Mongolia in 1999 (isolates F11 and F12) and in the Kulunda Steppe of Russia in 2002 (isolates F13 and F18), locations set about 2500 km apart [33] (Fig 3). All four strains share a large dsRNA fragment approximately 6 kb in size and smaller dsRNA molecules ranging from 1.2 kbp to 2 kbp (Fig 4A). PCR detection with virus-specific primers derived from the obtained sequences later in the study confirmed the presence of three viruses in the four *S*. *alkalinus* isolates. There were no detected phenotypic manifestations or growth rate differences for the strains containing dsRNA bands, compared to the virus-free isolates. All strains readily produced fruiting bodies and showed intense sporulation on the AA agar medium. Horizontal transfer of the viruses by anastomoses contact was tested in triplicates on the isolates F13 and F18 with the dsRNA-free isolate F7, but no transferred dsRNA was detected in the recipient strain after visualizing total nucleic acids content on a gel. No cured isolate was obtained from the 124 subsequent cultures generated from hyphal tips of the isolate F13 treated with cycloheximide during 4 passages. Therefore, we cannot compare the phenotype of an infected isolate with its isogenic uninfected counterpart. All viruses could be transmitted vertically by asexual (conidia) and sexual (ascospores) spores. Virus-like particles of 35–40 nm in diameter were detected in *S*. *alkalinus* strain F13 extract using transmission electron microscopy after negative staining (Fig 5).

### Novel fusarivirus

**Fig 6:**
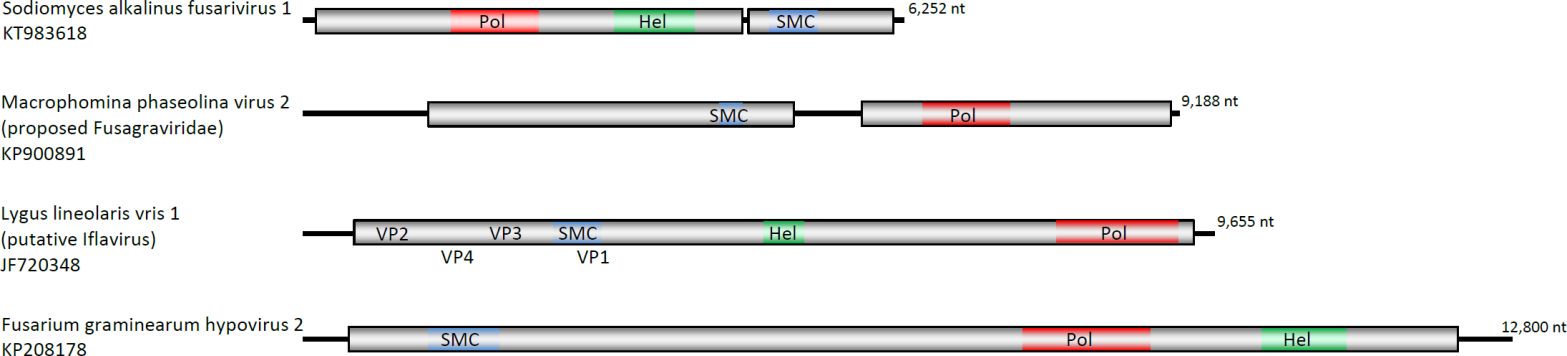
The genomic organization of SaFV1 compared to other viruses that possess structural maintenance of chromosomes domain. Boxes and lines represent open reading frames (ORFs) and non-coding sequences, respectively. Positions of polymerase (Pol, red), helicase (Hel, green), and structural maintenance of chromosomes (SMC, blue) domains are indicated. VP1-4 are structural proteins. Genomes are shown to scale.

The longest dsRNA segment isolated from *S*. *alkalinus* was 6240 nt long, excluding the poly(A) 3´-terminal tail, and had a GC content of 48.9%. Two ORFs were found on the coding strand of this RNA. The ORF1 encodes a putative polyprotein of 1526 aa (estimated mass of 172 kDa). An RdRp and a helicase motif were identified on this putative polyprotein (Fig 6). PSI-BLAST of the complete ORF1 polyprotein shows 50% and 48% aa identity, respectively, to Fusarium poae fusarivirus 1 and Rosellinia necatrix fusarivirus 1 as the most closely related viruses. The cDNA probe specific for the region of ORF2 was used in Northern blotting of the separated RNAs from the virus-free isolate F7 and SaFV1-containing isolate F13. Band corresponding to SaFV1 genome-size and faint band < 4kb indicating the presence of subgenomic RNAs were detected (Fig 4B).

**Fig 7:**
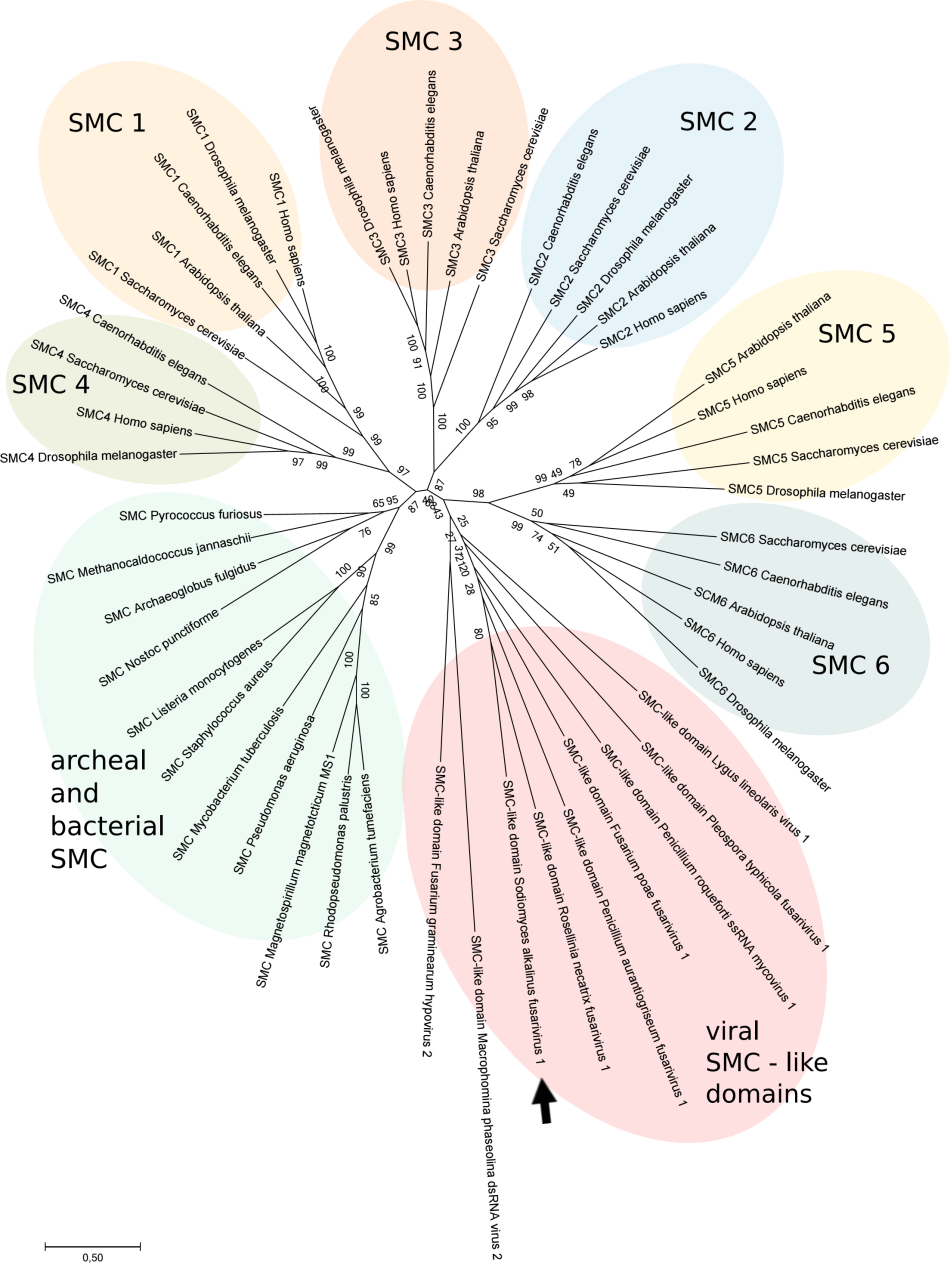
Phylogenetic tree of selected members of prokaryotic, eukaryotic, and viral SMC domains. Phylogenetic ML-tree with Poisson correction with 1000 replications generated from SMC and SMC-like amino acid sequences. sequences (all used organisms and accession numbers of the sequences are shown in S3 Table). Bootstrap values are given at the nodes.

**Fig 8:**
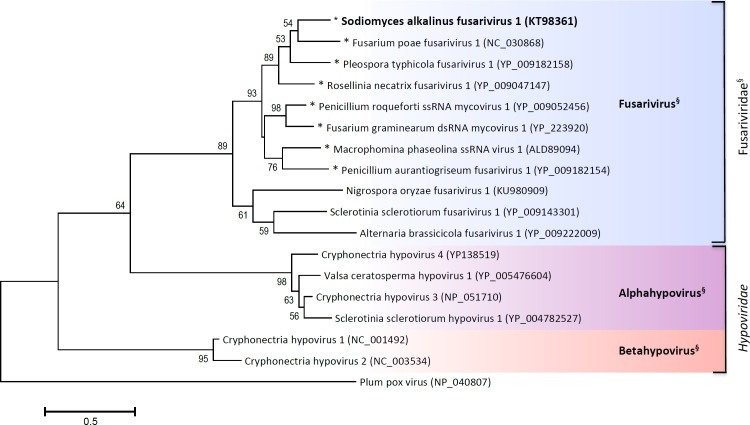
Phylogenetic tree as inferred from the I-VII RdRp motifs of fusariviruses. Maximum likelihood tree generated using RdRp segments of motifs I to VII with 1000 bootstrap replicates. Plum pox virus was used as an outgroup. Presence of the SMC domain in ORF2 protein of fusariviruses is indicated with an asterisk. Bootstrap values above 50% are given next to the branches. Section marks (§) indicate proposed taxa.

The ORF2 starts 69 nt downstream from the end of ORF1 at a nucleotide 4659. It ends with the UAA codon at a nucleotide 6182 and encodes a protein 507 aa long with an estimated mass of 55 kDa. The most similar proteins shared 27–30% aa identity with homologous proteins of other putative fusariviruses (E values from 6e^−10^ to 1e^−40^) (Table 1). No other proteins with significant similarity have been found in GenBank. The function of this protein remains unknown. However, a bacterial SMC-like (structural maintenance of chromosomes) domain (GenBank conserved domain TIGR02168) was found in a PSI-BLAST search in the N-half of the ORF2 protein of SaFV1. This domain was detected in similar positions on the ORF2 (ORF4 in case of FgV1) protein in all other fusariviruses with the exception of AbFV1, SsFV1, and NoFV1 (Table 1). In addition to fusariviruses, the SMC domain has been found in a predicted capsid protein 1 of Lygus lineolaris virus 1 (putative Iflavirus), which infects the tarnished plant bug [39]; in the N-part of the Fusarium graminearum hypovirus 2 polyprotein [40]; and in the C-terminal part of the ORF1 of Macrophomina phaseolina virus 2 (MpRV2 –KP900891, proposed Fusagraviridae) [41] (Fig 6). In order to reveal how the viral SMC proteins are related to bacterial and eukaryotic SMC´s, a maximum-likelihood tree was constructed (Fig 7). On this tree, the viral SMC formed a separated cluster with the closest relatives being the eukaryotic SMC5 and SMC6 subfamily proteins. The presence of the SMC domain across members of one lineage of the putative Fusariviridae family indicates its origin from a common ancestor and supports the phylogenetic relationships as inferred from the RdRp sequence (Fig 8).

**Table 1:** Presence of SMC domain in members of putative Fusariviridae family. Position of the SMC domain on the ORF2 and E-value is presented.

virus	position (aa)	E-value
Alternaria brassicicola fusarivirus 1	-	-
Fusarium poae fusarivirus 1	80–277	3.36e-08
Fusarium graminearum dsRNA mycovirus 1	25–176	8.88e-03
Macrophomina phaseolina ssRNA virus 1	49–266	7.36e-09
Nigrospora oryzae fusarivirus 1	-	-
Penicillium aurantiogriseum fusarivirus 1	35–234	9.32e-05
Penicillium roqueforti ssRNA mycovirus 1	42–282	1.11e-07
Pleospora typhicola fusarivirus 1	91–247	1.92e-06
Rosellinia necatrix fusarivirus 1	50–231	1.86e-05
Sodiomyces alkalinus fusarivirus 1	62–236	2.09e-07
Sclerotinia sclerotiorum fusarivirus 1	-	-

**Fig 9:**
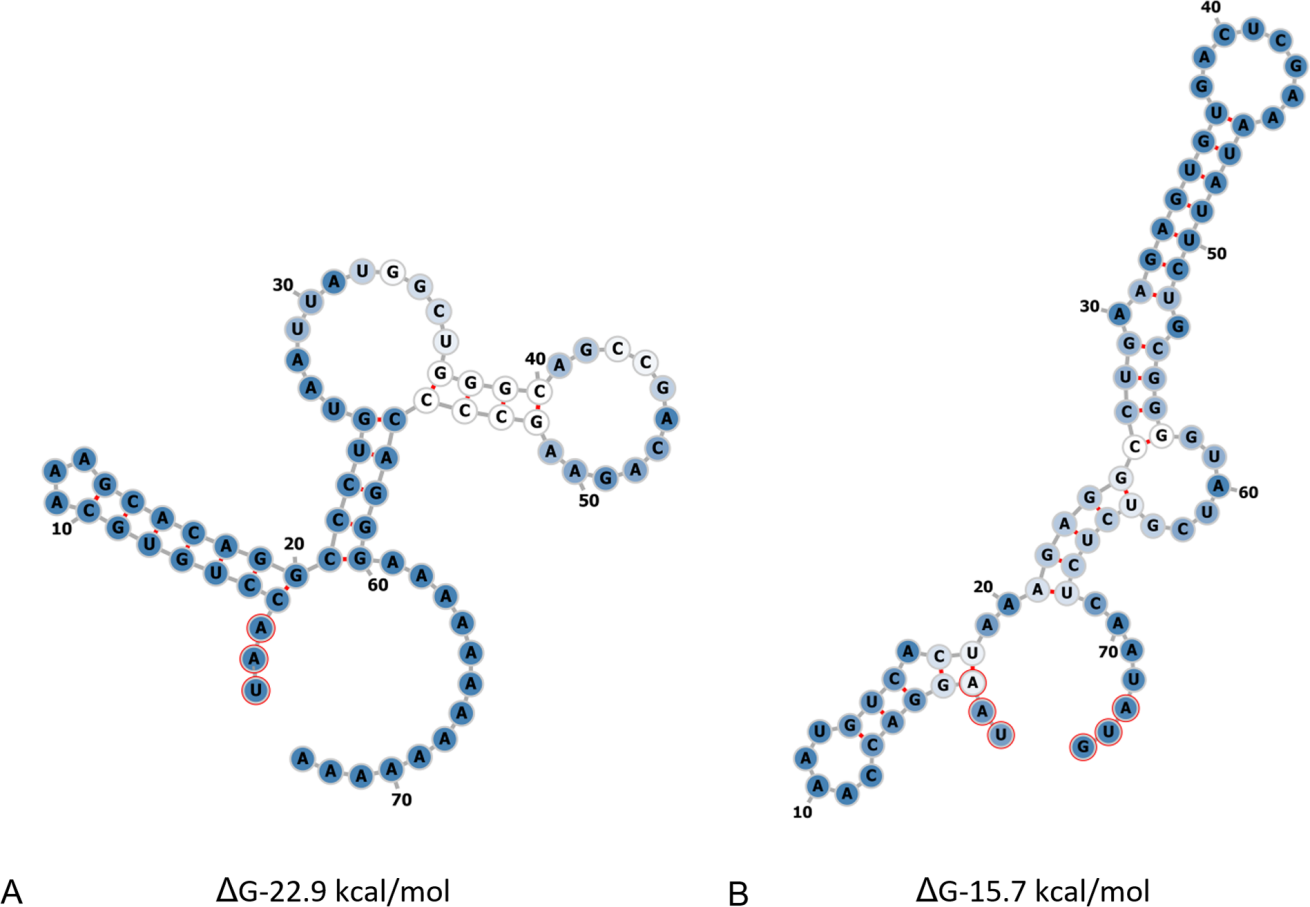
Potential secondary structures of 3´end, and ORF1-ORF2 intergenic region of Sodiomyces alkalinus fusarivirus 1 (SaFV1). a) Potential secondary structures of 3´end (from 6183–6239 nucleotides) and b) ORF1-ORF2 intergenic region (from 4590 to 4659 nucleotides). The ΔG values (kcal/mol) were calculated using RNAfold program. UAA stop codons and AUG codon are marked.

The ORF1–ORF2 intergenic region of SaFV1 is 69 nt long and is able to fold several stem-loop structures of ΔG −15.7 kcal/mol at 20°C. The 3´ UTR of SaFV1 is 57 nt long and formed a highly stable structure with a calculated ΔG of −22.91 kcal/mol at 20°C (Fig 9).

The presence of viruses in a fungus from an extreme environment led us to screen the sequence variability and evaluate potential selective forces in the evolution of the different viruses. Almost complete nt sequences of SaFV1 from four *S*. *alkalinus* isolates (F11, F12, F13, and F18) were obtained and compared. The viral sequences from the isolates F11 and F12 were identical and genetically distinct from those of the isolates F13 and F18, which were closely related to one another than to the sequences in the isolates F11 and F12. The aa sequences of ORF1 shared 95.6–97.8% sequence identity, and the aa sequences of ORF2 shared 93.3–97.4% identity between the isolates (Table 2).

**Table 2:** Amino acid sequence identities (in %) of ORF1 (under the diagonal) and ORF2 (above the diagonal) between the SaFV1-containing isolates of *S*. *alkalinus*.

ORF1 ORF2	F11	F12	F13	F18
F11		100	93.3	94.3
F12	100		93.3	94.3
F13	95.6	95.6		97.4
F18	96.6	96.6	97.8	

Synonymous and nonsynonymous nucleotide substitution analyses for the ORF1 and ORF2 were conducted according to the Nei-Gojobori model and using Jukes-Cantor correction [38]. The Pi(a)/Pi(s) ratio was 0.0147 for the polyprotein encoded on ORF1 and 0.0248 for protein encoded on ORF2. A substitution ratio less than 1 signifies a high degree of conservation of both ORFs and indicates strong purifying selection acting on these fusariviruses.

Phylogenetic analysis of the RdRp I-VII domains (a segment about 300 aa long) separates a cluster of the proposed genus Fusarivirus from two hypovirus clades (proposed Alphahypovirus and Betahypovirus genera) (Fig 8). All known fusariviruses are from the ascomycetous fungi, and we found no correlation between the nature and habitat of the host and the virus’ position on the tree.

### Novel betapartitivirus

**Fig 10:**
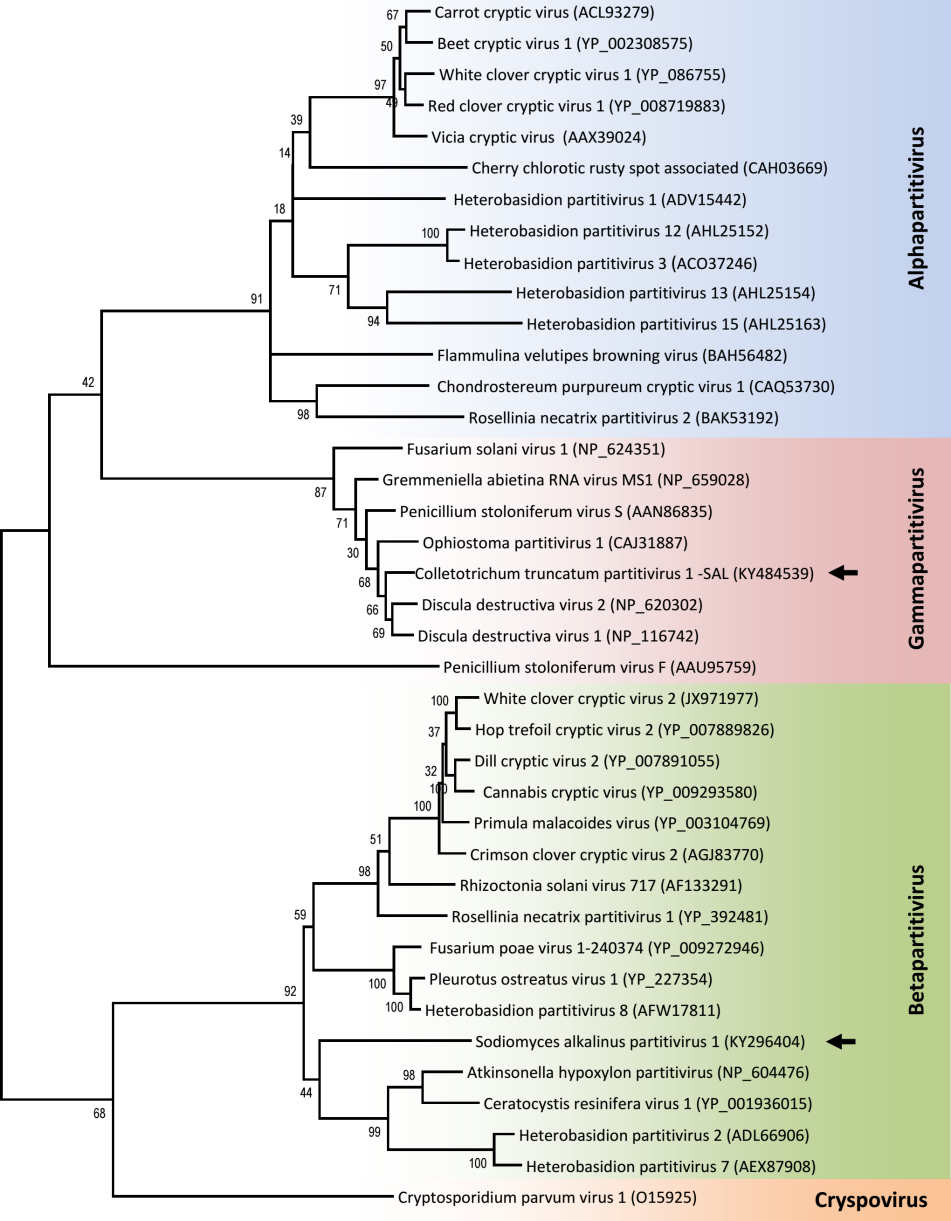
Phylogeny of SaPV1 and CtParV1-SAL in relation to other partitiviruses. ML phylogenetic tree generated from the RdRp amino acid sequence with 1000 bootstrap replicates.

**Fig 11:**
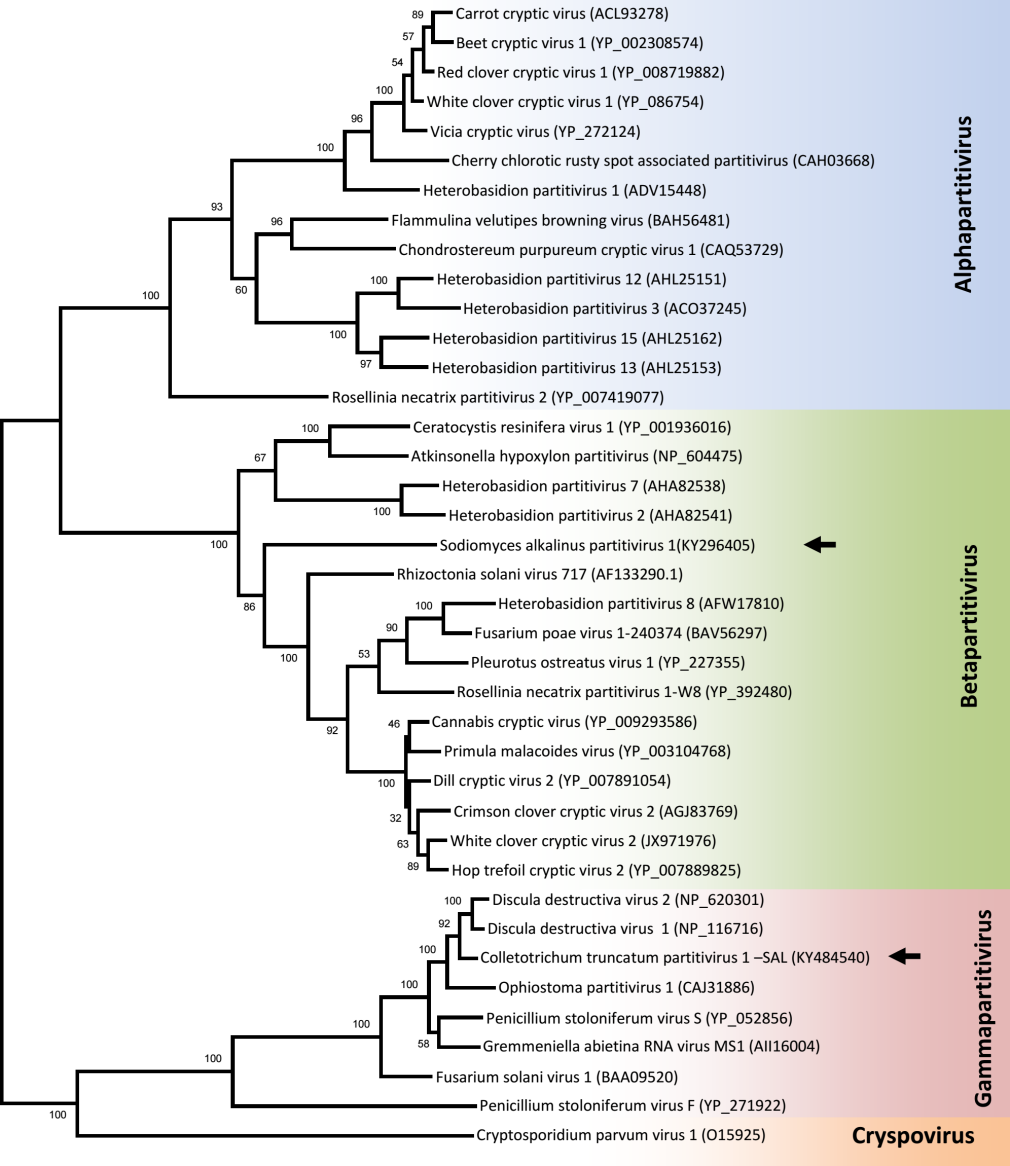
Phylogeny of SaPV1 and CtParV1-SAL in relation to other partitiviruses. ML phylogenetic tree generated from the CP amino acid sequence with 1000 bootstrap replicates.

The four smaller dsRNA bands visible in dsRNA preparations of the F11, F12, F13, and F18 strains of *S*. *alkalinus* (see Fig 4) could represent one quadripartite virus or, less commonly occurring, two bipartite viruses. Our sequence analyses showed two different RdRp genes present in these four segments, and thus multiple infections with two viruses were confirmed. The larger segments of about 2.4 kbp and 2.2 kbp belong to a new betapartitivirus, while the smaller 1.8 kbp and 1.5 kbp bands belong to a gammapartitivirus, which we will discuss in the next section (Figs 10 and 11).

The largest segment of 2402 nt contains one ORF and encodes a protein 753 aa long and with estimated mass of about 88 kDa. The RdRp domains typical for dsRNA viruses were found in this protein. The most similar sequences are the RdRps of Ustilaginoidea virens partitivirus 2 and Fusarium poae partitivirus 2 (both of genus *Betapartitivirus*) with 76% and 67% aa identity, respectively. The 5´ UTR is 67 nt long, while the 3´ UTR is 73 nt long. There are 38 As (76%) in the last 50 nt on the 3´ UTR, a feature commonly observed in betapartitiviruses. The smaller segment is 2122 nt long and contains one ORF encoding a CP protein 641 aa long of about 71 kDa. The noncoding regions are 50 nt and 146 nt long on the 5´and 3´ends, respectively. The last 50 nt on the 3´ UTR contains 14 As (28%). We conclude that these two dsRNA segments from *S*. *alkalinus* represent a novel virus in the genus *Betapartitivirus*, for which the name Sodiomyces alkalinus partitivirus 1 (SaPV1) is proposed. The 400 bp long amplified segment of the CP gene of SaPV1 from the isolates F11, F12, F13, and F18 was compared and analyzed for sequence polymorphisms. Five positions were variable among the isolates, and were nonsynonymous substitutions.

### Gammapartitivirus

The smallest dsRNA segments of 1.8 and 1.5 kbp contain one ORF each encoding the RdRp and CP proteins, respectively. Amino acid sequences of the encoded RdRp and CP share high identity (92% for RdRp and 85.9% for CP) with the corresponding genes of Colletotrichum truncatum partitivirus 1 (CtParV1) (genus *Gammapartitivirus*), hence, we regard this virus as a new strain (SAL–for *S**odiomyces* *al**kalinus* host) of CtParV1. The nucleotide sequences differed with the 80% and 77.1% respective identity with the corresponding RdRp and CP genes of CtParV1. Conversely, the 400 bp segments of the CP gene from the isolates F11, F12, F13, and F18 of *S*. *alkalinus* were 97.8–99.7% identical. This is a very rare example of presence of similar partitiviruses in such diverse hosts and environments. The nucleotide sequence variability between CtParV1 from *C*. *truncatum* from an unidentified crop in the U.S.A. [42] and CtParV1-SAL from *S*. *alkalinus* obtained from a soda soil environment in Asia [33] could not be explained by the adaptation to different codon-usage in the hosts, as the partitiviruses did not follow the [XYG+XYC] frequencies of their hosts (Table 3).

**Table 3:** Comparative codon-usage analysis of viruses from *S*. *alkalinus*. Frequency of [XYG + XYC] of *S*. *alkalinus* was computed from codon frequencies of RPB2 and TEF1-alpha protein coding genes sequenced previously [1], data for *Colletotrichum* sp. were from http://www.kazusa.or.jp.

virus genes	[XYG + XYC] (%)	hosts	[XYG + XYC] (%)
SaFV1 ORF1	54.6	*Sodiomyces alkalinus*	74.6
SaFV1 ORF2	49.9		
SaPV1 CP	66.3		
SaPV1 RdRp	56.5		
CtParV1-SAL CP	53.9		
CtParV1-SAL RdRp	51.1		
CtParV1 CP	57.4	*Colletotrichum sp*.	60–85
CtParV1 RdRp	54.5		

In previous studies, the equilibrium of different viruses in mixed infections has not been convincingly evaluated. Here, we performed relative quantification of the RdRp segment of the three (SaFV1, SaPV1, CtParV1-SAL) viruses in the isolate F13 of *S*. *alkalinus*. qRT-PCR analysis detected all three viruses in this isolate. SaPV1 as well as CtParV1-SAL were present in similar quantities, while SaFV1 was present only at about 1/20^th^ concentration. Furthermore, comparative codon-choice analysis showed significant differences in frequency of [XYG+XYC] in both RdRp and CP ORFs of these viruses, which support the hypothesis that there is no strong competition for the cellular resources between them (Table 3).

Newly generated viral sequences were deposited in GenBank with accession numbers KT983618, KY817860-61 for Sodiomyces alkalinus fusarivirus (SaFV1), KY296404-5, and KY484539-40 for Sodiomyces alkalinus partitivirus 1 (SaPV1), and Colletotrichum truncatum partitivirus 1 –isolate SAL (CtParV1-SAL), respectively.

## Discussion

The virosphere of extremophiles remains largely understudied, with the exception of archaea and some extremophilic (thermophilic, halophilic) bacteria, for which up to 80 complete viral sequences are known [43]. The present study reports the first case of mycoviruses present in an obligate alkalophilic filamentous fungus *Sodiomyces alkalinus*. *Acremonium alcalophilum*, the ex-type strain CBS 114.92, a closely related alkalophile, does not have detectable titers of dsRNA viral bands on agarose gel. The recently described alkalophilic *Sodiomyces magadii* and *Sodiomyces tronii* species [44] also seem to be free of viral bands, as is an unrelated facultative alkalophile *Emericellopsis alkalina* (Grum-Grzhimaylo, unpublished data). This makes *S*. *alkalinus* thus far the only filamentous alkalophilic fungus known to harbour dsRNA viruses.

There are several examples where the virus presence confers benefit to its host. For instance, a unique three-way plant–fungal–virus symbiosis has been described between a tropical panic grass from geothermal soils, *Dichanthelium lanuginosum*, the fungus *Curvularia protuberata*, and Curvularia thermal tolerance virus (CThTV) that confers heat tolerance to the grass host [45]. In a coastal dune grass environment, the fungus *Fusarium culmorum* confers salt tolerance to plants [46]. More than one putative mycovirus has been found in that salt habitat-adapted fungus, however, neither have their roles in this system been determined nor have the viruses yet been identified [11]. This finding might point to another example of a mutualistic relationship within a partitivirus–plant pair [25]. However, such mutualistic scenario between the viruses and *S*. *alkalinus* seems unlikely, since the fungus is not in symbiosis with other organisms and viruses were detected only in 4 out of 18 isolates. The fact that not all isolates of *S*. *alkalinus* have the viruses indicates that they are not involved in the adaptations to high salinity or high pH.

It is noteworthy that the three viruses were present in all four isolates together. Viral co-infections by distantly related or congeneric viruses have been described relatively often in different hosts [3, 47–50] but co-infection with different partitiviruses is known to occur in only a few hosts. Betapartitivirus and alphapartitivirus hosts have been described in a single strain of *Helicobasidium mompa* [3]. Penicillium stoloniferum virus F and S (both gammapartitiviruses) have been shown to co-infect a *Penicillium stoloniferum* strain and their coincident expression has been demonstrated in the host [51–52]. Extremely complex mixture of 16 different alpha- and betapartitivirus species were detected in two isolates of mycorrhizal *Ceratobasidium* fungus associated with *Pterostylis* orchids, recently [53].

Co-infection by two or more viruses may lead to complex interactions between viruses resembling synergism in plants and animals, enhancing fitness of the viruses, or antagonism, where the presence of one virus lowers the fitness of the others [54]. In the present study, it seems that the SaPV1 and CtParV1-SAL exhibited similar rates of expression in *S*. *alkalinus* as seen from similar intensities of the dsRNA bands on the agarose gel and from qRT-PCR data. However, there are cases where the viruses are replication-competent and their frequencies are biased towards one virus over the other. For instance, the co-infection of radish (*Raphanus sativus*) by Raphanus sativus cryptic virus 1 and 2 (putative alphapartitivirus and deltapartitivirus) showed a significantly larger amount of dsRNA from the former virus [55]. The co-infection of Shiitake mushroom (*Lentinula edodes*) by Lentinula edodes mycovirus HKB (proposed genus Phlegivirus) and Lentinula edodes partitivirus 1 (LePV1, betapartitivirus) increased replication of the RdRp gene of LePV1 by almost 6-fold in comparison to the infection of LePV1 alone [56]. An unpredictable effect was observed in the co-infection of *Rosellinia necatrix* by Rosellinia necatrix megabirnavirus 2 (RnMBV2) and Rosellinia necatrix partitivirus 1 (betapartitivirus). Specifically, double infection reduced mycelial growth down to 50–60% of what observed in single infections, and RnMBV2 was able to confer hypovirulence with the aid of a co-infecting partitivirus, while the individual viruses exhibited asymptomatic infections [57].

Observations of the *Heterobasidion parviporum* infection with distinct partitiviruses suggested that co-infections by distantly related viral species are more stable than those caused by conspecific strains. Furthermore, mutual exclusion was observed in the same host in closely related HetRV6 strains [58]. Long-term stable co-infection by different viruses could be due to little intracellular competition exemplified by different needs for genus-specific conserved 5´- and 3´-terminal genomic plus strand sequences that are thought to be involved in RdRp recognition for replication [25]. The different compartmentalization of partitiviruses could also play a role. On the contrary, in the complex multiple infections of *Ceratobasidium* mentioned above [53] it is suggested that RdRp segments of the putative distinct partitivirus species shares CPs. Here, we described a unique system of stable co-infection of an extremophilic fungus with two different partitiviruses and one fusarivirus. The viruses have no apparent phenotypic effect on its host and they do not appear responsible for the alkalophilic physiology of the fungus. The viruses do not have noticeable detrimental effect either, and the three viruses display stable coexistence and clonal transmission. This is of interest, because coexistence of different viruses might result in within-host competition between the viruses over replication and transmission, resulting in increased virulence at the fitness costs to the host. The origin of these viruses is unclear, as no other host is known for the described fusarivirus and betapartitivirus, but a related isolate from distant hosts exists for the gammapartitivirus. What balancing interactions between the three viruses and their host lead to their stable persistence remains an open question.

## Supporting information

S1 Table Primers used in this study. (DOCX)

S2 Table Next generation sequencing summary data. (DOCX)

S3 Table Organisms, their SMC domains and accession numbers used in Fig 7. (DOCX)
