# Supplementary material for: The alkalophilic fungus Sodiomyces alkalinus hosts beta- and gammapartitiviruses together with a new fusarivirus
Source: PLoS One. 2017 Nov 29;12(11):e0187799. doi: 10.1371/journal.pone.0187799 (PMC5706713; doi:10.1371/journal.pone.0187799)
Supplement: S1 Table — (DOCX) [file pone.0187799.s001.docx]

**Table S1.** **Primers used in this study**

| primer | Use | 5´-3´sequence |
| --- | --- | --- |
| SaFV 275R | 5 RACE | CCAAACTGGTGTATAAGAGC |
| SaFV 206R | 5 RACE | ATGACACATATTACTGGGCA |
| SaFV 84F |  | CCTCTTTGTGATTCTGTGTG |
| SaFV 1248R |  | AAACTTGAATATGTCCCCCA |
| SaFV 1194F |  | CATATCCCGGTACTTCAAGA |
| SaFV 2040R |  | GCCGAAAACACTCAATTCAT |
| SaFV 1996F |  | CTGCTTGCGTGATTTTGTAT |
| SaFV 3062R |  | TCAAACTTCATCACAAACCC |
| SaFV 2964F |  | CATTAATAGGTGGTCGTTCC |
| SaFV 3684R |  | GTGTTGCTTAAACTCATGGA |
| SaFV 3586F |  | TCTACCCACATCCCTCTC |
| SaFV 4349R |  | CTGGCTGTGTCAATGAATG |
| SaFV 4288F |  | GTGTCTGCCATGGAAATG |
| SaFV 5086R |  | GGTCATCCATTTGTTTGTCA |
| SaFV 5006R |  | TGGACCTTAAAAATGCTTGG |
| SaFV 5803R |  | CCGTTAAACCAGCATCTATC |
| SaFV 5696F |  | TGTCACTCATCATGGAATCT |
| Oligo(dT) |  | GGCCACGCGTCGACTAGTACTCGAGTTTTTTTTTTTTTTTTT |
|  |  |  |
| SaPV1 CP 424R | 5 RACE | TTGTTCATGATGTGTTGAGC |
| SaPV1 CP 410R | 5 RACE | TTGAGCTGCGTTGGAGGAC |
| SaPV1 CP 405F |  | GCTCAACACATCATGAACAA |
| SaPV1 CP 804R |  | CATCTATGCGTTCGTTAAGC |
| SaPV1 CP 785F |  | GCTTAACGAACGCATAGATG |
| SaPV1 CP 1720R |  | CTAGCGTCGGTGGTCTGC |
| SaPV1 CP 1703F | 3 RACE | GCAGACCACCGACGCTAG |
| SaPV1 CP 1815F | 3 RACE | TTACACGAATCAACTCTGTTCAACAT |
| SaPV1 Pol 553R | 5 RACE | TGATTCTTCAAATAGGATGTTAGAC |
| SaPV1 Pol 360R | 5 RACE | AGGTCAAAGTCTTCATCTCGTTGC |
| SaPV1 Pol 529F |  | GTCTAACATCCTATTTGAAGAATCA |
| SaPV1 Pol 1159R |  | GTGAAGAAAGCGGTCATCTA |
| SaPV1 Pol 1140F |  | TAGATGACCGCTTTCTTCAC |
| SaPV1 Pol 2068R |  | CTTCTTGAATATCTTCATTATATGGTG |
| SaPV1 Pol 1145F | 3 RACE | CACCATATAATGAAGATATTCAAGAAG |
| SaPV1 Pol 1146f | 3 RACE | CAACTTCCTGACTTTGACATTGAAGATGTG |
|  |  |  |
| SaPV2 CP 429R | 5 RACE | AGAGGAACTCCGCATACTTTG |
| SaPV2 CP 1175R | 5 RACE | GACGACAGAGAAGTAGCGAG |
| SaPV2 CP 409F |  | CAAAGTATGCGGAGTTCCTCT |
| SaPV2 CP 1181R |  | AGCAGCATTTCTATTGACCA |
| SaPV2 CP 1162F | 3 RACE | TGGTCAATAGAAATGCTGCTAC |
| SaPV2 CP 1248F | 3 RACE | CATCTCGCTTTGTCTGCTC |
| SaPV2 Pol 463R | 5 RACE | TTGGTTTGGTGAGAGGCT |
| SaPV2 Pol 298R | 5 RACE | CGAGTTAGACGAGTAAGTTGAG |
| SaPV2 Pol 277F |  | CTCAACTTACTCGTCTAACTCG |
| SaPV2 Pol 1004R |  | GGAGGATTTGAAAAGCAGCAC |
| SaPV2 Pol 984F |  | GTGCTGCTTTTCAAATCCTCC |
| SaPV2 Pol 1623R |  | TGATCCTTGGAGAACCACCC |
| SaPV2 Pol 1428F | 3 RACE | CTTTCCGTGAAACTAACGAATGGT |
| SaPV2 Pol 1489F | 3 RACE | ACTTGATGTTTCATTTACTCGTCTC |
| AAP |  | GGCCACGCGTCGACTAGTACGGGIIGGGIIGGGIIG |
| AUAP |  | AAGCAGTGGTATCAACGCAGAGTACGCGGG |
