# Supplementary material for: The alkalophilic fungus Sodiomyces alkalinus hosts beta- and gammapartitiviruses together with a new fusarivirus
Source: PLoS One. 2017 Nov 29;12(11):e0187799. doi: 10.1371/journal.pone.0187799 (PMC5706713; doi:10.1371/journal.pone.0187799)
Supplement: S2 Table — (DOCX) [file pone.0187799.s002.docx]

**Table S2.** **Next generation sequencing summary data**

| Reference sequence | consensus length | total read count | average coverage |
| --- | --- | --- | --- |
| SaPV1 CP | 2066 | 285 | 12,6 |
| SaPV1 RdRp | 2362 | 297 | 11,9 |
| SaPV2 CP | 1458 | 315 | 19,6 |
| SaPV2 RdRp | 1627 | 415 | 22,2 |
| SaFV1 | 6223 | 14181 | 224,1 |
