# Supplementary material for: The alkalophilic fungus Sodiomyces alkalinus hosts beta- and gammapartitiviruses together with a new fusarivirus
Source: PLoS One. 2017 Nov 29;12(11):e0187799. doi: 10.1371/journal.pone.0187799 (PMC5706713; doi:10.1371/journal.pone.0187799)
Supplement: S3 Table — (DOCX) [file pone.0187799.s003.docx]

**Table S3: Organisms, their SMC domains and accession numbers used in Fig.7.**

| **Organism** | **Type of SMC domain** | **Accession number** |
| --- | --- | --- |
| *Agrobacterium tumefaciens* | Bacterial | AKC06499.1 |
| *Rhodopseudomonas palustris* | Bacterial | WP_047308236.1 |
| *Magnetospirillum magnetotacticum MS1* | Bacterial | KIL99688.1 |
| *Pseudomonas aeruginosa* | Bacterial | KFL13993.1 |
| *Mycobacterium tuberculosis* | Bacterial | CCE38378.1 |
| *Staphylococcus aureus* | Bacterial | KFL08011.1 |
| *Listeria monocytogenes* | Bacterial | AJA83513.1 |
| *Nostoc punctiforme* | Archaeal | ACC84041.1 |
| *Archaeoglobus fulgidus* | Archaeal | WP_048095927.1 |
| *Methanocaldococcus jannaschii* | Archaeal | WP_010871167.1 |
| *Pyrococcus furiosus* | Archaeal | CAD66602.1 |
| *Saccharomyces cerevisiae* | SMC1 | KZV11593.1 |
| *Arabidopsis thaliana* | SMC1 | AAS68515.1 |
| *Caenorhabditis elegans* | SMC1 | NP_001040658.2 |
| *Drosophila melanogaster* | SMC1 | NP_651211.2 |
| *Homo sapiens* | SMC1 | AAI12128.1 |
| *Caenorhabditis elegans* | SMC2 | AAC47834.1 |
| *Saccharomyces cerevisiae* | SMC2 | KZV11633.1 |
| *Drosophila melanogaster* | SMC2 | AAD52673.1 |
| *Arabidopsis thaliana* | SMC2 | NP_201047.1 |
| *Homo sapiens* | SMC2 | AA44164.1 |
| *Drosophila melanogaster* | SMC3 | AAF48625.2 |
| *Homo sapiens* | SMC3 | NP_005436.1 |
| *Caenorhabditis elegans* | SMC3 | NP_001129842.1 |
| *Saccharomyces cerevisiae* | SMC3 | KZV10199.1 |
| *Saccharomyces cerevisiae* | SMC3 | KZV10199.1 |
| *Drosophila melanogaster* | SMC4 | NP_723996.1 |
| *Homo sapiens* | SMC4 | NP_005487.3 |
| *Saccharomyces cerevisiae* | SMC4 | KZV09332.1 |
| *Caenorhabditis elegans* | SMC4 | NP_497935.1 |
| *Arabidopsis thaliana* | SMC5 | OAO95282.1 |
| *Homo sapiens* | SMC5 | CAC39247.1 |
| *Caenorhabditis elegans* | SMC5 | NP_494935.1 |
| *Saccharomyces cerevisiae* | SMC5 | KZV07849.1 |
| *Drosophila melanogaster* | SMC5 | NP_001262176.1 |
| *Saccharomyces cerevisiae* | SMC6 | KZV09633.1 |
| *Caenorhabditis elegans* | SMC6 | NP_496476.2 |
| *Arabidopsis thaliana* | SMC6 | AAD54770.1 |
| *Homo sapiens* | SMC6 | CAC39248.1 |
| *Drosophila melanogaster* | SMC6 | NP_651228.1 |
| *Lygus lineolaris virus 1* | Viral SMC-like domain | JF720348 |
| Pleospora typhicola fusarivirus 1 | Viral SMC-like domain | NC_028470 |
| Penicillium roqueforti ssRNA mycovirus 1 | Viral SMC-like domain | NC_024699 |
| Fusarium poae fusarivirus 1 | Viral SMC-like domain | NC_030868 |
| Penicillium aurantiogriseum fusarivirus 1 | Viral SMC-like domain | NC_028467 |
| Rosellinia necatrix fusarivirus 1 | Viral SMC-like domain | NC_024485 |
| Macrophomina phaseolina dsRNA virus 2 | Viral SMC-like domain | KP900891 |
| Fusarium graminearum hypovirus 2 | Viral SMC-like domain | KP208178 |
| Sodiomyces alkalinus fusarivirus 1 | Viral SMC-like domain | KT98361 |
